# Temporal and Spatial Comparisons of Underwater Sound Signatures of Different Reef Habitats in Moorea Island, French Polynesia

**DOI:** 10.1371/journal.pone.0135733

**Published:** 2015-09-09

**Authors:** Frédéric Bertucci, Eric Parmentier, Laëtitia Berten, Rohan M. Brooker, David Lecchini

**Affiliations:** 1 USR 3278 CNRS-EPHE-UPVD, CRIOBE, Moorea, French Polynesia; 2 Laboratoire de Morphologie Fonctionnelle et Evolutive, Université de Liège, Liège, Belgium; 3 School of Marine Science and Policy, University of Delaware, 19958, Lewes, Delaware, United States of America; 4 Laboratoire d'Excellence "CORAIL", Moorea, French Polynesia; Leibniz Center for Tropical Marine Ecology, GERMANY

## Abstract

As environmental sounds are used by larval fish and crustaceans to locate and orientate towards habitat during settlement, variations in the acoustic signature produced by habitats could provide valuable information about habitat quality, helping larvae to differentiate between potential settlement sites. However, very little is known about how acoustic signatures differ between proximate habitats. This study described within- and between-site differences in the sound spectra of five contiguous habitats at Moorea Island, French Polynesia: the inner reef crest, the barrier reef, the fringing reef, a pass and a coastal mangrove forest. Habitats with coral (inner, barrier and fringing reefs) were characterized by a similar sound spectrum with average intensities ranging from 70 to 78 dB re 1μPa.Hz^-1^. The mangrove forest had a lower sound intensity of 70 dB re 1μPa.Hz^-1^ while the pass was characterized by a higher sound level with an average intensity of 91 dB re 1μPa.Hz^-1^. Habitats showed significantly different intensities for most frequencies, and a decreasing intensity gradient was observed from the reef to the shore. While habitats close to the shore showed no significant diel variation in sound intensities, sound levels increased at the pass during the night and barrier reef during the day. These two habitats also appeared to be louder in the North than in the West. These findings suggest that daily variations in sound intensity and across-reef sound gradients could be a valuable source of information for settling larvae. They also provide further evidence that closely related habitats, separated by less than 1 km, can differ significantly in their spectral composition and that these signatures might be typical and conserved along the coast of Moorea.

## Introduction

Ambient sea noise is largely composed of sounds generated by abiotic sources (geophony), such as wind and waves, and biotic sounds (biophony) produced by various marine organisms [1–3]. However, a third source, sounds produced through human activities (anthrophony), is increasingly common, especially in coastal marine environments [4–6]. Together, geophony, biophony and anthrophony combine to create the acoustic signature of an environment. Such signatures, or soundscapes, provide a set of acoustic cues that can influence many aspects of a marine organism’s behaviour, including mating, feeding activity, predator or prey detection, orientation and territory defence [2, 4–9]. Indeed, sounds have an advantage over other forms of sensory information, such as visual or olfactory cues, as they propagate in all directions independent of factors such as light, flow, or turbidity, allowing them to spread over a considerable distance while still providing accurate directional information [10, 11].

Data regarding the nature of marine soundscapes now exists for many locations globally, including sites within the Pacific [12–15], Atlantic [16, 17] and Indian Oceans [18–20], allowing preliminary descriptions of variation in acoustic activity between and within environments to be made. For example, in Australian waters, the combined vocal activity of fishes and crustaceans is most intense at dusk, with an increase of 20 dB above the mean ambient noise level [21, 22]. However, the biotic noise signature can also vary over longer temporal scales due to interspecific differences in vocal behaviour, with the vocal activity of some marine organisms increasing during certain seasons [13, 23, 24], while that of others appears to remain consistent throughout the year [25–27].

Although the key features of the acoustic signatures that distinguish some marine ecosystems have been identified, localized acoustic variability between adjacent habitats, i.e. those separated by less than 1 km, has rarely been characterized. On coral reefs for instance, how the soundscape of an inner reef crest differs from that of a barrier reef, and how temporally variable these soundscapes are is largely unknown. However, recent evidence suggests that spectral differences between spatially associated reef habitats can be largely due to variation in the sonic activity of marine organisms, i.e. soniferous fishes and snapping shrimps [26]. Another recent study comparing the soundscapes of a temperate urchin-dominated rocky reef to a sandy beach identified significantly higher sound amplitude in frequencies between 800 Hz and 2500 Hz on the rocky reef as well as diel variations in the temporal and spectral composition of these soundscapes [25]. As acoustic cues are known to influence the behaviour of many fish and invertebrate larvae at settlement [27–30], understanding how habitat soundscapes vary over small spatial scales could help explain the distribution of marine organisms. Although there is little empirical evidence of how soundscape variability can affect settlement, [31] showed the larvae of some fish species are preferentially attracted to, or repelled by, the sounds of different coral reef habitats. At least 65% of species tested selected sounds from preferred habitats, validating the hypothesis that sound is used to select their settlement sites. However, the acoustical characteristics of these habitats were not precisely described in this study, and no estimation of diel variability was performed.

The aim of this present study was to describe the temporal and spectral acoustic features of five different, but spatially associated, marine habitats surrounding Moorea Island, French Polynesia. Because many coral reef-associated species have highly specialized habitat requirements we hypothesize that biotic variation between habitat types will create unique acoustic signatures that could provide key information for larval marine organisms during settlement.

## Materials and Methods

### Sampling sites

The study was conducted from February 1^st^ to April 30, 2010 and 2011 at Moorea Island (French Polynesia; 17°31' S; 149°51' W). Moorea has a large lagoon composed of five main habitat types: 1) the inner reef crest (CR), characterized by breaking waves, strong water flux, and a substratum comprised mainly of coral rubble with less than 10% live coral; 2) the barrier reef (BR), characterized by a water depth of 1–5 m and a substratum comprised of up to 30% live coral from a diverse range of species; 3) the fringing reef (FR) characterized by a water depth of 1–2 m and a substratum comprised of between 10–20% live coral from a limited number of species; 4) the pass (PA), a coral free area located in front of an opening in the reef crest, and canalizing the water flow to the ocean; and 5) the coastal mangrove forest (MG), comprised of a mud-sand substratum covered with mangrove trees located along the shoreline with a depth less than 1 m [32].

**Fig 1:**
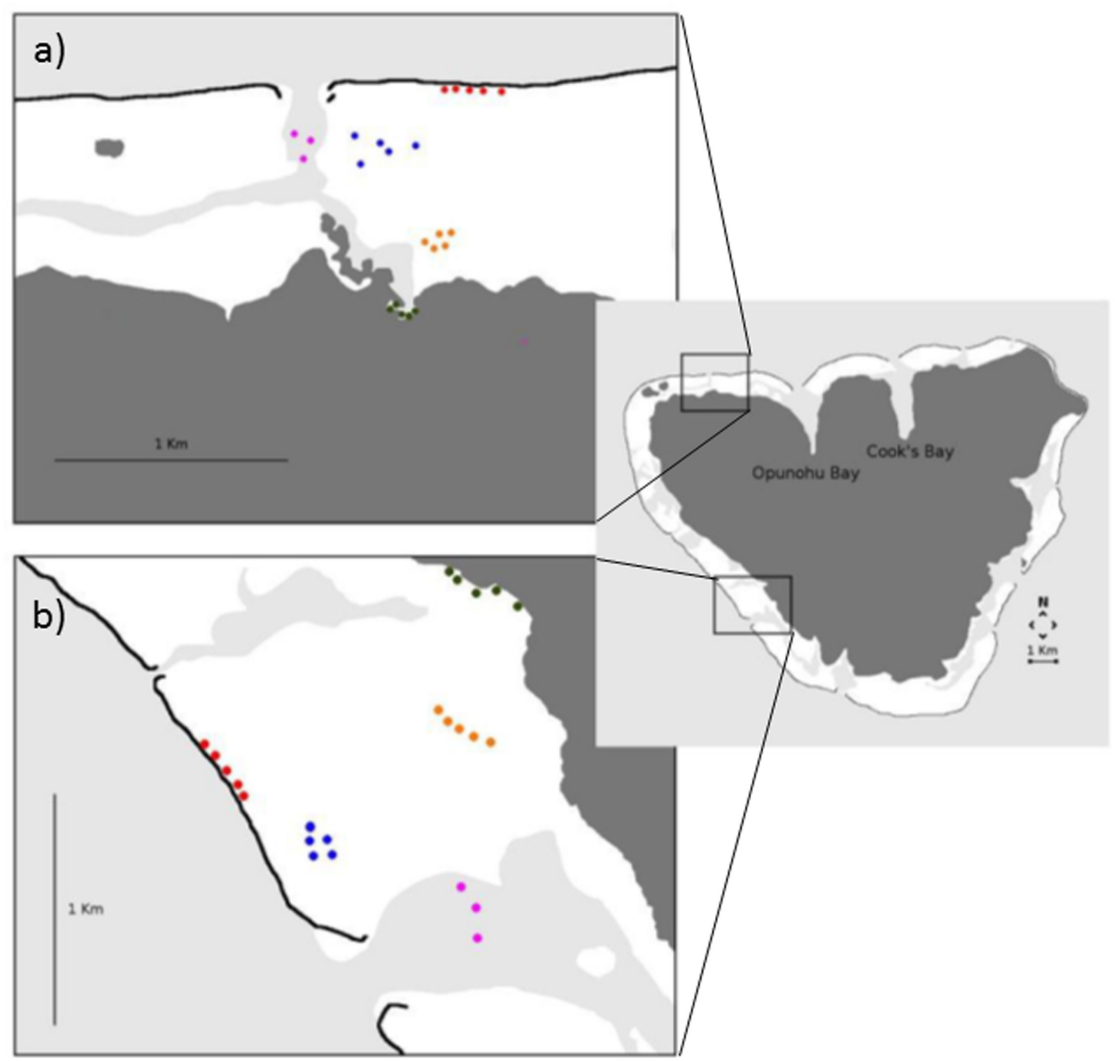
Maps of the North (a) and West (b) coasts of Moorea Island. Locations of the sound recordings for each of the five habitats (red dots = reef crest; blue dots = barrier reef; purple dots = pass; orange dots = fringing reef and green dots = mangrove forest). Maps drawn by the authors from an aerial photograph of Moorea taken by the CRIOBE in 2008 from a private plane. For representative purposes only.

Five GPS points were taken for RC, BR, FR and MG habitats, whilst only three GPS points were taken for PA habitat due to safety restrictions. In order to assess spatial and temporal variations, the study was repeated in two similar sites; one located on the North coast of Moorea and one located on the West coast (Fig 1). These sites were selected since they are known settlement sites [33] and their soundscapes have been shown to attract fish larvae [31].

### Recordings

Sounds were recorded from kayak using a HTI-96-MIN wide-band omni-directional hydrophone (sensibility -165 dB re, High Tech Inc, USA) connected to a calibrated Tascam DR-07 portable digital recorder (frequency response 20 to 20 KHz +1/3 dB re, TEAC America, Inc.). Sounds were digitized at 44.1 kHz (16-bit resolution). Replicate recordings at each GPS point were collected on the same day to control for potential between-day effects. At each GPS point, 1 to 3 recordings were taken so that the number of replicates varied between 5 and 15 for each habitat (Table 1). Recordings were taken when wind speed was lower than 5 knots to prevent the kayak from drifting and to reduce wind and sea surface turbulences. Recordings were 60 sec in duration, taken at a depth of half the water column. Recording were conducted between 11:00 and 14:00 for day recordings (two different adjacent locations could be recorded in the same day), from 17:30 to 18:30 for dusk recordings (only one location per day due to the reduced time window) and from 20:00 to 22:00 for night recordings (again, only one location a day due to the reduced time window). The tide at Moorea is very weak (10 cm). Recordings were conducted during the week of new moon as this is the period of highest larval recruitment [33, 34].

**Table 1:** Intensities and corresponding frequencies of local maxima and minima for habitats recorded at the North coast in 2011.

		Sound intensity				
		Local max.	Frequency	Local min.	Frequency	Mean intensity(20–5000 Hz)
		(dB re 1μPa.Hz^-1^)	(Hz)	(dB re 1μPa.Hz^-1^)	(Hz)	(dB re 1μPa)
CR	Day	90 ± 6	172	72 ± 5	947	78 ± 6
	Dusk	93 ± 10	172	73 ± 9	1034	80 ± 7
	Dawn	85 ± 4	172	70 ±4	689	78 ± 7
BR	Day	89 ± 6	172	69 ± 5	947	78 ± 6
	Dusk	78 ± 3	172	60 ± 2	861	74 ± 8
	Dawn	80 ± 3	172	65 ± 4	1034	76 ± 8
PA	Day	90 ± 5	172	85 ± 3	1034	88 ± 5
	Dusk	92 ± 1	258	88 ± 4	861	93 ± 5
	Dawn	88 ± 5	258	85 ± 4	689	91 ± 7
FR		84 ± 12	172	64 ± 11	1120	70 ± 11
MG		87 ± 12	172	69	> 1000	70 ± 12

CR = reef crest; BR = barrier reef; PA = pass; FR = fringing reef and MG = mangrove forest. Values are mean ± SE. The last column shows the mean ± SE sound level (dB re 1 μPa.Hz-1) for the 20 to 5000 Hz frequency range.

Recordings were first cleaned by visually inspecting sonograms using Avisoft-SASlab Pro software (Avisoft Bioacoustics, Germany) to detect and remove any unwanted anthropogenic sound sources (e.g. boat engines, slapping waves or paddle shocks against the kayak) from the analysis. Power spectra (Fast Fourier Transform FFT, 512 points Hamming window, 86.13 Hz resolution, frequency bandwidth: 20–5000 Hz) were then created from the analysis of 5s samples for each sound file and averaged to present the spectrum of each environment.

### Data analysis

Characteristics of each habitat were described on the basis of frequency modulations of power spectra. Mean values and standard deviations of intensities were calculated for all sampled frequencies, (Fast Fourier Transform FFT, 512 points Hamming window, 86.13 Hz resolution, from 20 to 5000 Hz), for each sound file. Habitat sounds recorded on the North coast in 2011 were compared to each other (two-way ANOVA followed by Tukey's multiple comparison test). For each habitat, diel sound variations were determined by comparing mean intensities between day periods (day-dusk-night) for the North coast in 2010 and 2011, and for the West coast in 2010 (Two-way ANOVA followed by Tukey's multiple comparison test). Spatial variations within a given habitat type between each day period were determined between the North and West coasts in 2010 (Two-way ANOVA followed by Sidak's multiple comparison test). Finally, temporal variations within the same habitats and day periods were compared between both years for the North coast (Two-way ANOVA followed by Sidak's multiple comparison test). All analyses were performed using the GraphPad Prism 6 software (GraphPad Software, Inc. USA).

### Ethics statement

This study was conducted in accordance with the guidelines of the French Polynesia committee for publication ethics and no specific permissions were required for the recordings of the different habitats. Moreover, no organism was captured during the study. Thus, the recording experiments were approved by the CRIOBE animal ethics committee.

## Results

### Spectral description of the different habitats

The pass, characterized by an absence of corals and a rapid water flow, was the loudest habitat with a mean ± SD sound intensity of 91 ± 5 dB re 1μPa.Hz^-1^. The power spectrum was characterized by a local maximum of 89 ± 4 dB re 1μPa.Hz^-1^ found at lower frequencies of ca. 200 Hz, before a slight intensity decrease down to a local minimum of 86 ± 3 dB re 1μPa.Hz^-1^ at frequencies from 700 to 1000 Hz. Intensities did not differ significantly between 1000Hz and 5000Hz (Table 1). The inner reef crest, the barrier reef and the fringing reef, habitats characterized by the presence of corals, had lower mean sound intensities of 78 ± 7 dB re 1μPa.Hz^-1^ (CR), 76 ± 8 dB re 1μPa.Hz^-1^ (BR) and 70 ± 11 dB re 1μPa.Hz^-1^ (FR) respectively. These habitats showed local peaks at *ca*. 170 Hz for intensities of 89 ± 7 dB re 1μPa.Hz^-1^, 82 ± 4 dB re 1μPa.Hz^-1^ and 84 ± 12 dB re 1μPa.Hz^-1^ for CR, BR and FR respectively. These peaks were then followed by a continuous decrease of intensities until local minima of 72 ± 6 dB re 1μPa.Hz^-1^ at *ca*. 900 Hz for the CR; 65 ± 4 dB re 1μPa.Hz^-1^ at *ca*. 950 Hz for the BR and 64 ± 11 dB re 1μPa.Hz^-1^ at 1120 Hz for the FR. At higher frequencies, intensities increased up to a plateau of *ca*. 80 dB re 1μPa.Hz^-1^ for the CR and the BR. Intensities of the FR at higher frequencies were lower, at *ca*. 75dB re 1μPa.Hz^-1^ (Table 1). The mangrove forest had a mean intensity of 70 ± 12dB re 1μPa.Hz^-1^ with a local maximum of 87 ± 12 dB re 1μPa.Hz^-1^ at 170 Hz. Intensity then decreased to a plateau of ca. 69 dB re 1μPa.Hz^-1^ from 1000Hz to 5000Hz. Mangrove sounds did not vary significantly between sites (Table 1).

### Within-habitat diel variation in acoustic signatures

**Fig 2:**
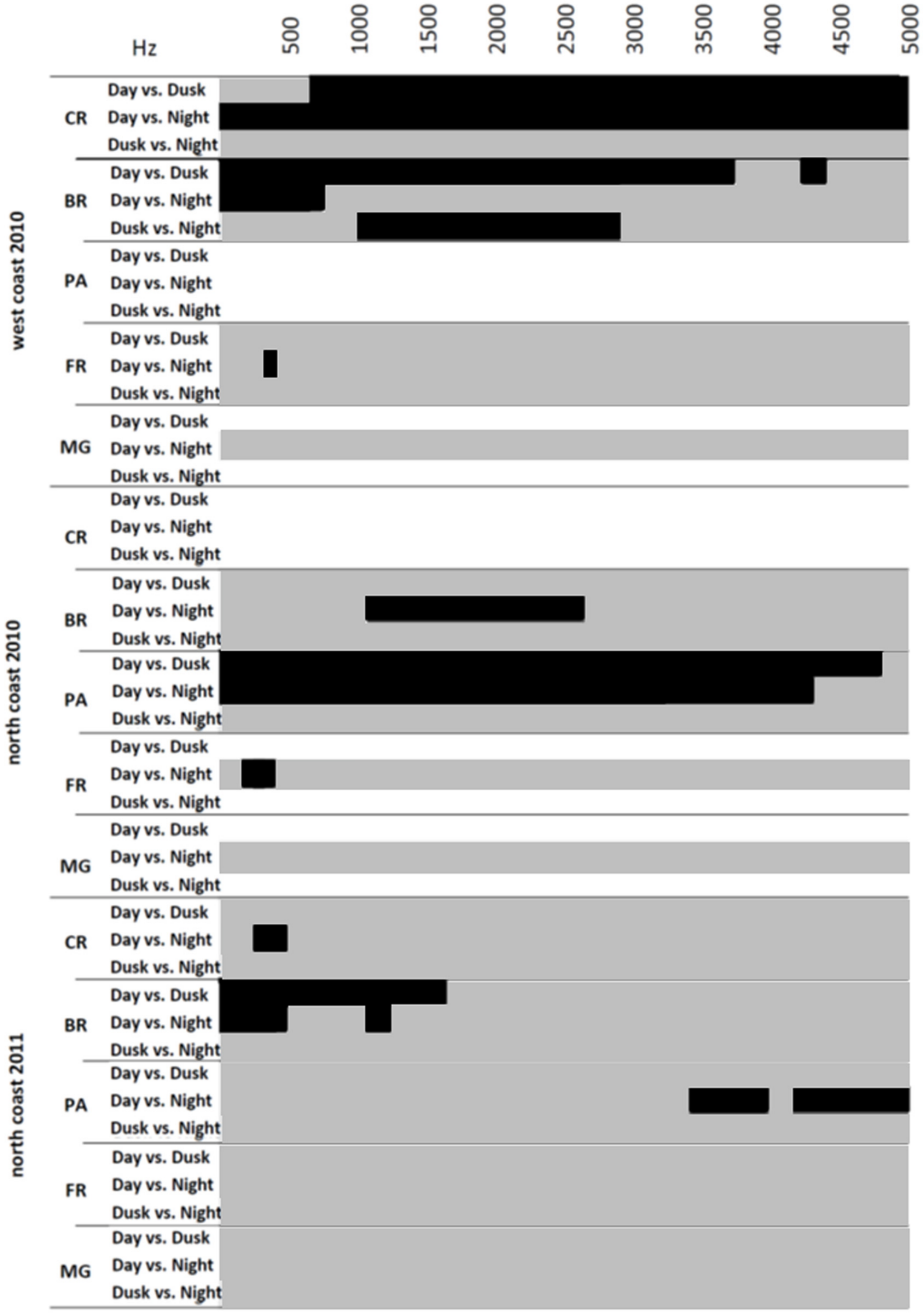
Comparison of sound intensities between day periods. Results of sampled frequencies (Fast Fourier Transform FFT, 512 points Hamming window, 86.13 Hz resolution, in the 20–5000 Hz range) at the West Coast in 2010 (top), the North Coast in 2010 (middle) and the North coast in 2011 (bottom). Black = P < 0.05. Grey = non-significant difference (Tukey’s multiple comparison post-hoc test). White = no data available.

No significant daily differences were observed in the mangrove forest recordings (n _2010_ = 30 and n _2011_ = 35 for either the North Coast and n _2010_ = 20 or the West Coast; F_2,511_ = 2; P > 0.05) (Fig 2).

Likewise, fringing reef recordings showed little daily variation. In 2010, at both North and West coast sites, the sound of fringing reef was significantly louder during the day than during the night for a short frequency range below 500 Hz (n _North_ = 30; n _West_ = 25; F_2,511_ = 62.00; P < 0.05). However, on the North Coast in 2011, no significant variation in intensity was observed for any frequency of the power spectrum between day periods (n = 35; F_2;511_ = 2; P > 0.05) (Fig 2). At the pass, recordings made on the North Coast in 2010 showed a significant intensity increase during dusk and night compared to the day (n = 25; F_2,511_ = 69.0; P < 0.05). These differences occurred for frequencies above 1600 Hz at dusk, and above 2100 Hz at night. There was no significant difference between sounds recorded at dusk and night (Fig 2). At the North Coast barrier reef site, sound intensity varied significantly between day and dusk for frequencies up to 4000 Hz in 2010, with higher intensities recorded during the day (n = 25; F_2,511_ = 69.00; P < 0.05). This difference was also identified in 2011 but only up to *ca*. 1700 Hz (n = 35; F_2,511_ = 63.0; P < 0.05) (Fig 2). Night recordings presented significantly lower intensities than day recordings for frequencies below 800 Hz at the West Coast in 2010 (n = 20; F_2,511_ = 72.0; P < 0.05), and for frequencies below 500 Hz at the North Coast in 2011. This difference was not found at the North Coast in 2010 where only frequencies between 1030 Hz and 2600 Hz differed between day and night. At the West Coast in 2010, significantly lower intensities were found at dusk for frequencies between 1000 Hz and 3000 Hz although no significant differences were identified between dusk and night at the North Coast in either year (Fig 2). Recordings from the West Coast inner reef crest in 2010 showed significantly higher sound intensities at dusk (starting from 600 Hz) and at night (entire spectrum) compared to the day (n = 25; F_2,511_ = 49.5; P < 0.05) with no difference observed between dusk and night. While no data is available for the North Coast in 2010, in 2011 intensity was significantly lower at night than during the day only for frequencies below 500 Hz (n = 35; F_2,511_ = 75.0; P < 0.05) (Fig 2).

### Between-habitat diel variation in acoustic signatures

No significant diel variation in sound intensity was identified between the inner reef crest and the barrier reef (n = 140; F_4,511_ = 2; P > 0.05), or between the fringing reef and the mangrove forest (n = 185; F_4,511_ = 2; P > 0.05), although during the day sound intensity tended to be lower at the fringing reef at frequencies between 250 Hz and 1800 Hz. During the day and night, sound levels were significantly higher at sites far from shore, the inner crest and barrier reef, than at those sites closest to shore, the fringing reef and mangrove forest (n = 20–45; F_4,511_ = 2; P > 0.05). This tendency also occurred at dusk, although differences were not significant (Fig 2).

**Fig 3:**
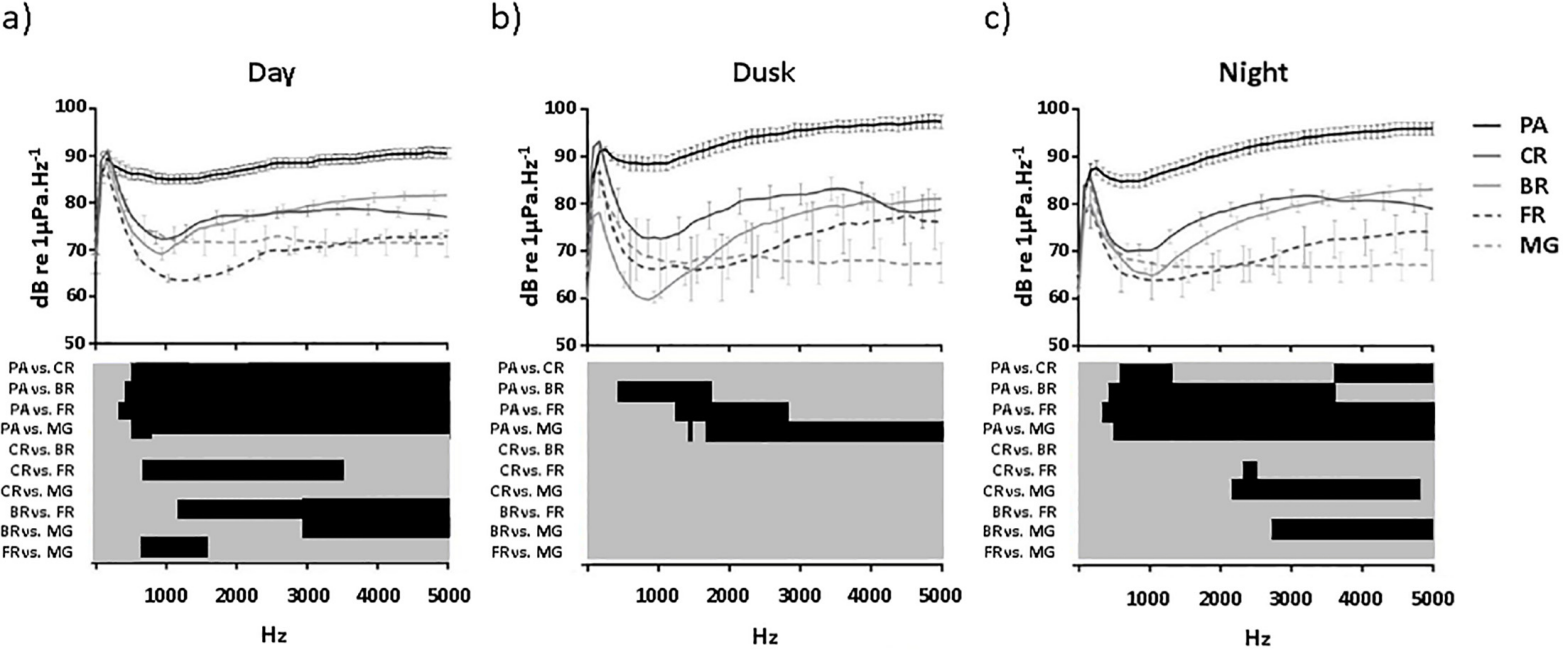
Average power spectra for the five habitats recorded at the North Coast in 2011. Recordings were performed during the day (a), at dusk (b) and at night (c). CR = inner reef crest; BR = barrier reef; PA = pass; FR = fringing reef and MG = mangrove forest. Values are mean ± SE for the 20 to 5000 Hz frequency range. Error bars are not shown for each frequency point for CR, BR, FR and MG but are staggered along the spectra lines in order to avoid overlapping lines and ensure greater clarity in their visualisation. All error bars are displayed for PA. Bottom: significant differences between habitats (multiple comparison post-hoc test); black = P < 0.05. Grey = non-significant difference.

During the day, the pass showed significantly higher sound intensities than all other habitats between frequencies of 500 Hz to 5000 Hz. Moreover, with the exception of the reef crest, which did not differ from the barrier reef and the mangrove forest, all habitats differed from each other at a certain range of their acoustic spectra (Fig 3a). At dusk, only the pass continued to be significantly different from other habitats, but only at a narrowed range of its total spectrum (Fig 3b). At night, the pass again presented the most different spectrum with higher sound intensities. At night the acoustic signature of the reef crest was not significantly different to either the barrier or fringing reef but differed between 2000 Hz to 5000 Hz with its most distant habitat, the mangrove forest. The barrier reef also differed from the mangrove forest at night with higher intensities between 2700 Hz and 500Hz, but showed a similar spectrum to the neighbouring fringing reef (Fig 3c).

### Comparison of acoustic signatures between North and West Coasts in 2010

**Fig 4:**
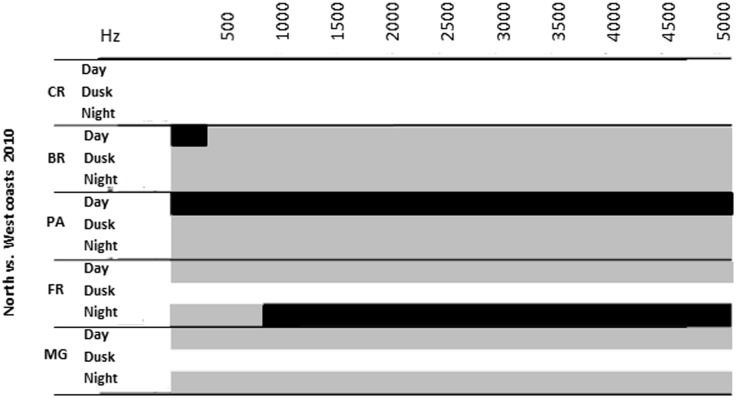
Comparison of sound intensities between the North and West Coasts in 2010. Results of sampled frequencies (Fast Fourier Transform FFT, 512 points Hamming window, 86.13 Hz resolution, in the 20–5000 Hz range). Black = P < 0.05. Grey = non-significant difference (Tukey’s multiple comparison post-hoc test). White = no data available.

Sound acoustic signature of the mangrove forest did not differ between North and West Coasts at any diel period in 2010 (n _North_ = 30; n _West_ = 20; F_2,511_ = 1.90; P > 0.05). Sounds of the barrier reef did not differ significantly between dusk and night although sound level was significantly higher at the North Coast during the day at frequencies below 500 Hz (n _North_ = 25; n _West_ = 20; F_2,511_ = 48.0; P < 0.05) (Fig 4).

At the pass, sound levels were higher during the day at the North Coast for the entire frequency range (n _North_ = 25; n _West_ = 15; F_2,511_ = 48.0; P < 0.05) but there were no differences between sites at dusk and night (Fig 4). At the fringing reef, the level of frequencies above 800 Hz were significantly higher at night on the West Coast (n _North_ = 30; n _West_ = 35; F_2,511_ = 70.0; P < 0.05) (Fig 4).

## Discussion

Numerous studies have now shown that the ambient sounds produced by reefs can attract larval fishes and/or crustacean, suggesting that reef noise could play an important role during settlement [30, 35–38]. However, our understanding of how soundscapes vary between different reef habitats, and how these differences could affect settlement patterns, is extremely limited. To this end, in this study we have characterized the acoustic features of five common, spatially linked, reef habitats and have described the specific spectral signatures of each.

In Moorea, reef habitats could be differentiated on the basis of their mean sound level alone. The pass had the highest sound level, most likely due to the rapid flow of water through the habitat that creates turbulence and friction against the substrate. In contrast, the relatively slow moving waters surrounding the coastal mangrove forest displayed the lowest sound level. Between these two habitats there was a declining gradient in sound intensity ranging from the inner reef crest (characterized by breaking waves and strong water flow), to the barrier reef (characterized by high coral density, species richness, and a depth of 1–5 m), and the fringing reef (characterized by low coral density and species richness and depth of 1–2 m). Therefore, it appears that high water flow and biodiversity-promoting coral cover may elevate mean sound levels with these factors resulting in louder and more frequent acoustic events. The pass, the inner reef crest and the barrier reef (i.e. the noisiest habitats) were further characterized by diel variations in sound intensity, with sound in the pass and the inner reef crest elevated at night, while the sound of the barrier reef was elevated during the day. The elevated nocturnal sound levels observed in habitats closest to the open ocean may help facilitate the detection of reefs by pelagic larvae, many of which approach and settle onto the reef at night. Only weak variations (i.e. at frequencies below 1000 Hz) occurred between day and night on the fringing reef while sound intensity in the mangrove forest did not vary at all. Thus, a spatial gradient of diel sound variation appears to exist, ranging from strong variations on the reef crest to little or no variation close to shore. The larvae of species from a variety of reef habitats appear to use reef noises to locate appropriate settlement sites. With this in mind, these results suggest larvae could potentially use the acoustic gradient that exists between the outer reef and shoreline to orientate themselves within the lagoon and promote movement towards or away from specific areas. Besides characterizing different habitats, acoustic variation may also be used by larvae to judge relative habitat quality as there is some evidence that sound intensity is greater on healthy reefs compared to those that are degraded [39].

The soundscape of the pass differed most substantially from other habitats, with the highest mean sound intensity (ca. 90 dB re 1μPa.Hz^-1^, 20–5000 Hz) likely due to the stronger water flow which can increase sound levels across a broad range of frequencies [40]. The pass collects waters from both the reef crest and the shore, bringing them to the ocean in a continuous water stream. Variation in the intensity of this water stream may explain the *ca*. 10 dB higher sound level found at the North Coast, where the pass is only ~180 m wide, creating stronger water flow than is found on the West Coast where the pass is wider at ~220 m [32, 41].

Habitats characterized by the presence of coral (*i*.*e*. the inner reef crest, the barrier reef, and the fringing reef) showed a similar spectral pattern with a local maximum peak at ca. 180 Hz decreasing to a local minimum between 690 Hz and 1035 Hz. This general pattern is similar to spectral curves recorded on reefs on the Great Barrier Reef, Australia [42], and Las Perlas Archipelago [43]. However, all five habitats exhibited significant differences in their specific frequency range. For example, the barrier reef had the highest intensities at frequencies above 2800 Hz, while the inner reef crest differed from the fringing reef as it had higher intensities at frequencies above 500 Hz during the day and for frequencies above 1300 Hz during the night. These differences suggest that associated larvae could respond to a specific set of frequencies and intensities that define their appropriate habitat. This is supported by evidence from choice experiments that have indicated that white noise is not attractive to larvae [31]. In Moorea, many reef fish larvae can discriminate between the soundscapes produced by barrier reef, fringing reef and mangrove habitats and exhibit a behavioural preference for the sounds of their preferred adult habitat [44]. It appears likely that the auditory abilities of fishes are finely tuned to the sounds produced by the most appropriate settlement location.

It is important to note that sound recorded in 2010 took place a few days after cyclone Oli passed through French Polynesia, coming within 250 Km of Moorea Island. Even though this event caused little disturbance to the lagoon (Lecchini, personal communication), it did increase the amount of coral debris and terrigenous inputs that may have resulted in some unrecognised change to lagoonal sound spectra, be it due to changes in biological activity [45] or via some other source.

Overall, these results highlight that, on coral reefs, variations in acoustic signatures can occur at small spatial scales due to habitat-specific biotic and abiotic factors. As many larvae have specialised habitat requirements, and use sounds to locate these habitats as they return to the reef, acoustic variations may help explain differential patterns of settlement. Although general similarities existed between habitats characterized by live coral, the presence of small but consistent spectral differences between these coral rich habitats suggest that larvae may be able to use these cues to orientate within heterogeneous environments.
